# Mycoplasma pneumoniae-Induced Rash and Mucositis (MIRM): A Case Report

**DOI:** 10.7759/cureus.84964

**Published:** 2025-05-28

**Authors:** Hamad A Shaheen, Moneerah A AlGassim, Mohanned M Alraddadi, Reem H Al-Ruwaili, Hamid A Yousef Mohammed

**Affiliations:** 1 Infectious Disease, Medical Speciality Department, King Fahad Medical City, Riyadh, SAU; 2 Allergy and Immunology, Medical Specialty Department, King Fahad Medical City, Riyadh, SAU; 3 Internal Medicine, Riyadh Second Health Cluster, King Fahad Medical City, Riyadh, SAU; 4 Medicine, Vision College, Riyadh, SAU; 5 Medicine, Ibn Sina University, Khartoum, SDN

**Keywords:** extra-respiratory complications, mirm, mycoplasma pneumoniae, mycoplasma pneumoniae-induced rash and mucositis (mirm), mycoplasma pneumonia infection

## Abstract

This case report describes a 27-year-old military serviceman in Saudi Arabia who developed severe mucocutaneous lesions - oral and genital ulcers, conjunctivitis, and eruptive rash - following an upper respiratory infection. Initially, his clinical presentation raised suspicion of Behçet’s disease; however, further evaluation and investigation favored Mycoplasma pneumoniae-induced rash and mucositis (MIRM) as a final diagnosis. His management included antibiotics and systemic corticosteroids, leading to marked clinical improvement. This case report highlights the diagnostic challenges of MIRM, particularly in regions where it is underreported. It underlines the importance of considering MIRM in patients with mucocutaneous eruptions, even without classic respiratory findings. By sharing this experience, we are aiming to enhance awareness of MIRM features and facilitate timely diagnosis. The patient’s recovery also supports MIRM’s generally favorable prognosis with appropriate treatment.

## Introduction

Mycoplasma pneumoniae infection occurs worldwide. It is a major cause of respiratory illness, particularly in children and young adults. Unlike other bacterial pathogens, Mycoplasma pneumoniae lacks a cell wall [1], which contributes to its unique clinical presentation and resistance to common beta-lactam antibiotics [2]. This pathogen often leads to a spectrum of respiratory symptoms, ranging from mild cough and fever to severe pneumonia, and can sometimes present with extrapulmonary manifestations.

Despite its frequent occurrence, the clinical diagnosis of Mycoplasma pneumoniae infection can be challenging due to its atypical symptoms and the overlap with other respiratory infections. An array of extrapulmonary manifestations may develop during M. pneumoniae infection [3,4]. The most significant are cold-agglutinin hemolytic anemia, arthritis, pericarditis, Guillain-Barré syndrome, and mucocutaneous manifestations.

Mucocutaneous eruptions, associated with M. pneumonia infection characterized by rashes affecting both mucous membrane and skin, include erythema multiforme, vesicles, bullous, petechiae, urticaria, Stevens-Johnson syndrome, or toxic epidermal necrolysis.

A recent distinctive form of mucocutaneous rash was reported in 2014, known as Mycoplasma pneumoniae-induced rash and mucositis (MIRM). MIRM is a rare disease, usually observed in children and adolescents. It is characterized by a prominent mucositis with single or a few scattered skin lesions, and urogenital lesions, which are quite common. Diagnosing MIRM can be challenging, however, a diagnosing criterion was proposed by Canavan et al. in 2015 [5], which states that the patient should have the following features: 1) Skin detachment <10% of the total body surface; 2) Suggestive lesions of at least two mucosal sites; 3) Scattered atypical targets; 4) Evidence of M. pneumoniae infection. 

To our knowledge, there were no similar case reports published in Saudi Arabia. In addition, the patient had atypical symptoms, which initially made the diagnosis of M. pneumoniae infection unlikely. Based on that, hopefully, we believe that this case report would contribute to a better understanding of MIRM.

## Case presentation

A 27-year-old male with no significant past medical history was referred to King Fahad Medical City (KFMC) for evaluation of suspected Behçet's disease. Ten days prior, he developed an upper respiratory tract infection after contact with colleagues who displayed similar symptoms. His condition progressed to include painless redness in both eyes, diffuse oral ulcers accompanied by odynophagia, and painless genital ulcers. He also experienced a high fever of 40°C for two days and bilateral elbow and knee pain, which was later compounded by a productive cough with whitish sputum. The patient, a smoker with a 10-pack-year history, works in the military.

**Figure 1:**
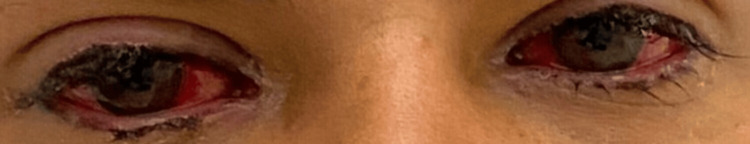
Bilateral follicular conjunctivitis with sticky blepharitis

**Figure 2:**
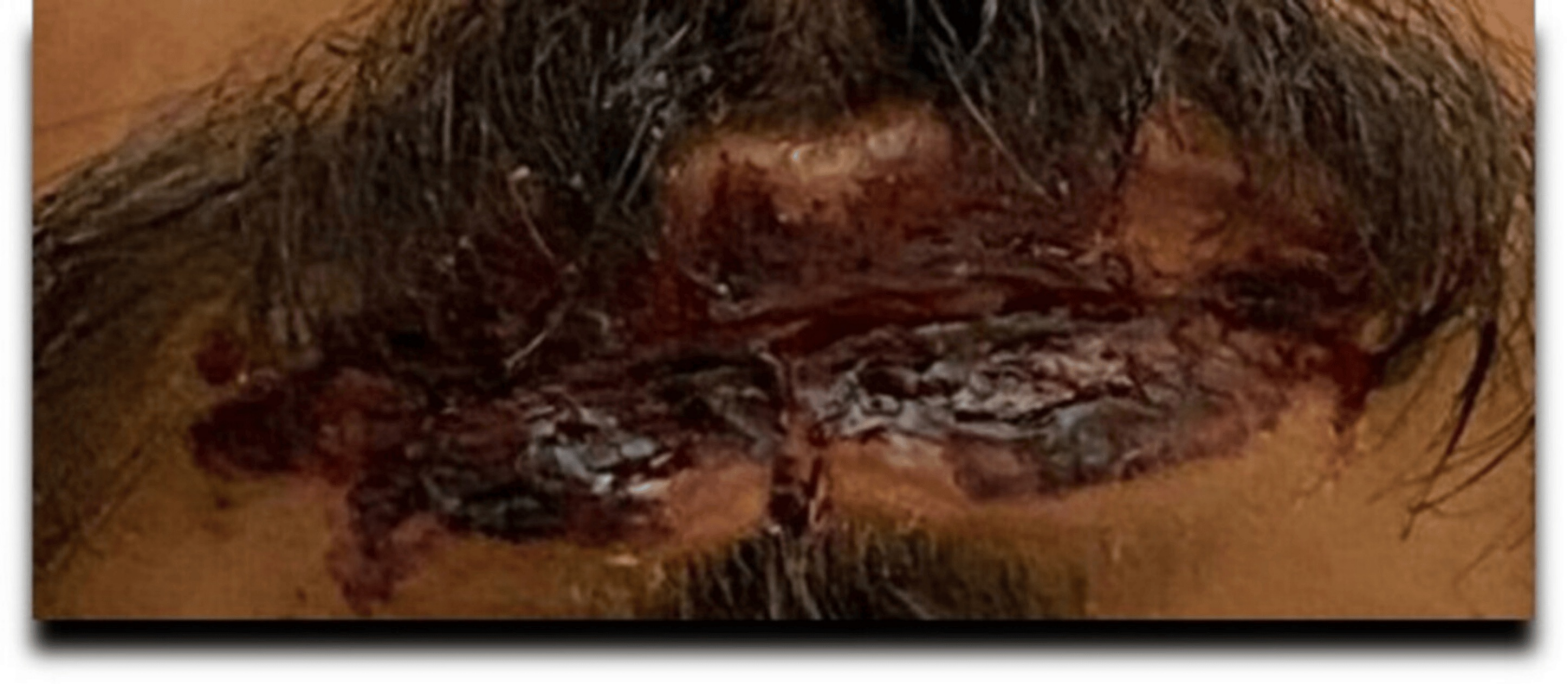
Ill-demarcated hemorrhagic crusts over the upper and lower lips

**Figure 3:**
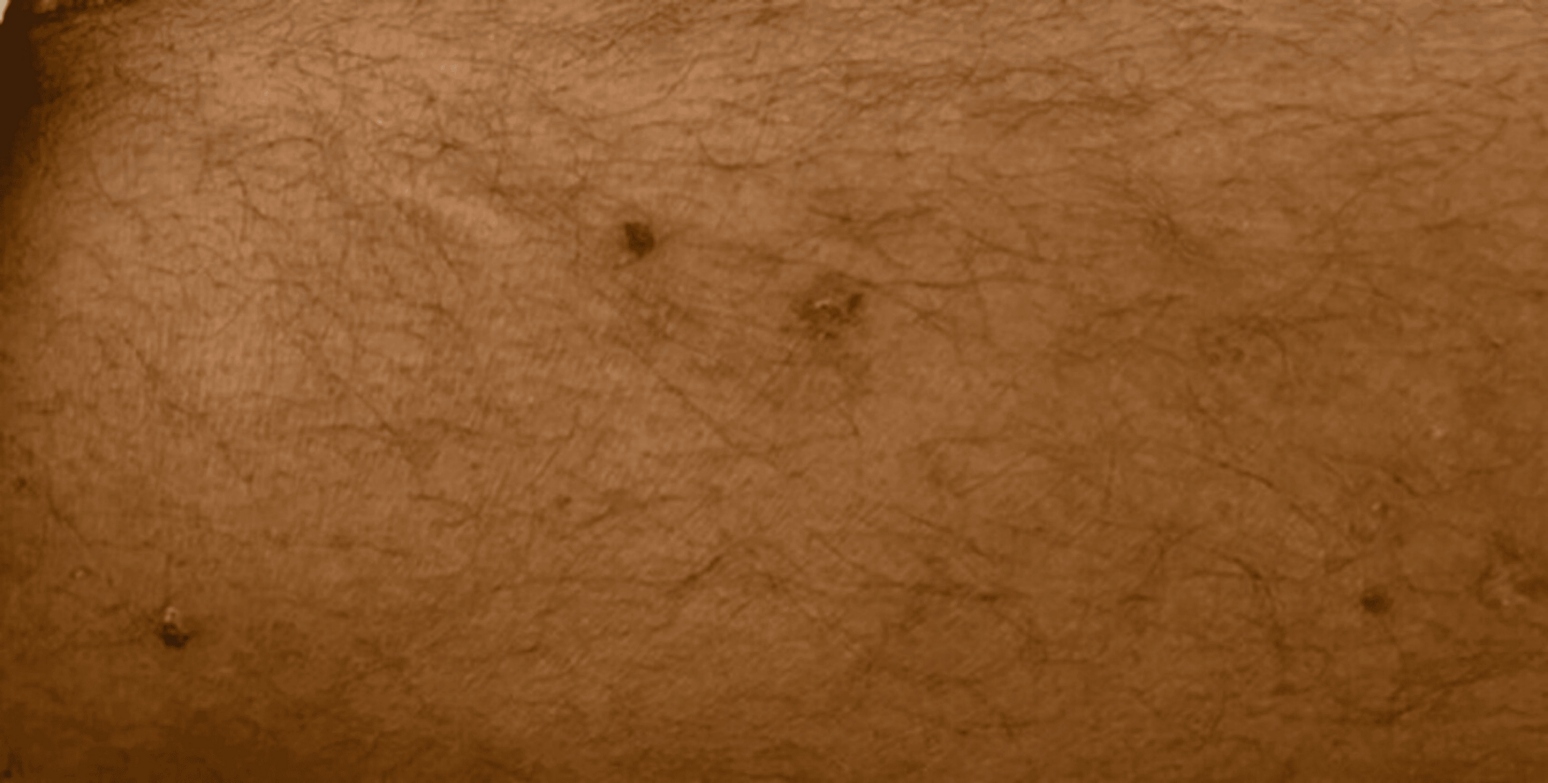
Multiple dry crusted plaques on the upper extremities.

In a previous admission to Alzulfi Hospital, he was treated with ceftriaxone, levofloxacin, and prednisolone. Upon examination at KFMC, his vital signs were stable, although his SpO2 was 93% on 2 liters of nasal cannula oxygen. The physical exam revealed bilateral red eyes (Figure 1). A skin examination showed ill-demarcated hemorrhagic crusts over the upper and lower lips (Figure 2), white spots in the buccal mucosa, multiple dry crusted plaques on the upper and lower extremities (Figure 3), and multiple erythematous targetoid ulcerated macules with scalloped borders on the scrotum and penis. The chest examination revealed equal airflow on both sides, absence of crackles or crepitations, and normal heart sounds.

Initial laboratory workup revealed a normal complete blood count (CBC), but elevated erythrocyte sedimentation rate (ESR) and C-reactive protein (CRP) levels (Table 1). Polymerase chain reaction (PCR) testing for HIV, a viral panel from nasopharyngeal swab (including adenovirus, coronavirus, influenza, enterovirus, etc.), and Methicillin-resistant Staphylococcus aureus (MRSA) screen were all negative. Cultures taken from the eye, blood, nasopharynx, and urine returned negative results, except for a dry swab wound culture from the penile ulcers, which showed heavy growth of extended-spectrum beta-lactamase (ESBL)-producing E. coli. Rheumatologic tests, including antinuclear antibody (ANA), antineutrophil cytoplasmic antibodies (ANCA), RF, C3/C4, cyclic citrullinated peptide (CCP), and double-stranded DNA (dsDNA) antibodies, were unremarkable, and screening for syphilis and gonococcus culture was also negative. A chest X-ray was unremarkable. Skin biopsy from scrotal lesions showing intracorneal and spinous neutrophilic dermatitis with no evidence of viral cytopathic changes seen. Grocott's methenamine silver (GMS) and Periodic acid-Schiff (PAS) special stains were negative for fungal elements.

**Table 1:** Laboratory Analysis Results With Normal Ranges ESR: erythrocyte sedimentation rate, CRP: C-reactive protein, ANA: antinuclear antibody, ANCA: antineutrophil cytoplasmic antibodies, CCP: cyclic citrullinated peptide, Ig: immunoglobulin, ESBL: extended-spectrum beta-lactamase, Abs: antibodies, CLIA: Clinical Laboratory Improvement Amendments, PCR: polymerase chain reaction, MRSA: Methicillin-resistant Staphylococcus aureus, HSV: herpes simplex virus, WBC: white blood cells, RBC: red blood cells, HPF: high-power field

Category	Labs	Result	Normal Range
Hematology	WBC	10.50 10/UL	3.90 - < 11.00
	Hematocrit	45%	37-52%
	Platelets	385 10 /UL	150-450
Inflammation & Autoimmune Markers	ESR	> 100 mm/h	0-20mm/h
	CRP	> 64.1 mg/L	<5.0 mg/L
	ANA	NEGATIVE	
	ANCA	NEGATIVE	
	CCP	NEGATIVE	
	RF	NEGATIVE	
	DNA AB	NEGATIVE	
	C3 C4	NEGATIVE	
Microbiological tests	Blood culture	No culture after 5 days	
	Mycoplasma pneumoniae. Abs. (IgG).	>200.00	<10.00 AU/ml
	Mycoplasma pneumoniae. Abs. (IgM) (CLIA)	>27	<10.00 AU/ml
	Acinetobacter screen	No Acinetobacter isolated	
	Wound culture (scrotum)	Heavy growth E. coli ESBL	
	Candida screening	No yeast isolate	
	Respiratory cultures	Scant growth, normal and respiratory flora (NEGATIVE)	
	Urine culture	No growth at 42 hours	
	Swab culture (mouth & penile)	NEGATIVE	
	Syphilis screen	NEGATIVE	
	Gonococcus	NEGATIVE	
	PCR HIV-1	Quantitative -> negative	
	PCR viral panel (nasopharynx)	Unremarkable	
	MRSA PCR	NEGATIVE	
	HSV PCR	NEGATIVE	
Urinalysis	WBC	22/HPF	
	RBC	443/HPF	
	Bacteria in urine	Positive	

During hospitalization, the patient remained vitally stable and showed improvement with continued intravenous antibiotics, including piperacillin/tazobactam (4.5 g) for three days, and meropenem (1 g) for eight days. He was also given oral prednisolone 20mg for five days. He also received hydrocortisone ointment twice/day for 14 days and mometasone ointment for crusted papules on the body twice/day for 14 days. The patient's clinical history did not meet the diagnostic criteria for Behçet's disease as outlined by the International Study Group (ISG) [6]. According to the ISG's established guidelines, a diagnosis requires the presence of recurrent oral ulcers along with two other major criteria (genital ulcers, eye involvement, skin lesions, or positive pathergy test). However, the patient's presentation lacked sufficient evidence of these criteria, particularly in terms of recurrent oral lesions and the other major findings required for diagnosis. An ophthalmologist confirmed severe bilateral follicular conjunctivitis with sticky blepharitis and recommended treatment with azithromycin (1 g) once, along with ofloxacin (0.3%) two drops daily for seven days and erythromycin ointment (0.5%) for seven days.

A subsequent consultation with a rheumatologist suggested that a rheumatologic cause was unlikely, favoring a viral etiology. However, viral screenings, including PCR for herpes simplex virus (HSV) swabs from oral and penile lesions and a comprehensive viral panel, returned negative results. Ultimately, as no definitive diagnosis was confirmed, testing for Mycoplasma pneumoniae (IgM and IgG) antibodies was conducted, which turned out positive, confirming Mycoplasma pneumoniae infection and specifically addressing the diagnosis of Mycoplasma pneumoniae-induced rash and mucositis (Table 1). During follow-ups, the patient exhibited significant improvement in skin manifestations, and the lesions healed.

## Discussion

Mycoplasma pneumoniae is recognized as one of the smallest free-living organisms and serves as a common bacterial pathogen responsible for infections in the upper respiratory tract and pneumonia. Notably, studies have demonstrated substantial variability in the prevalence of Mycoplasma pneumoniae, which may be linked to the limited availability of rapid and specific diagnostic methods for the disease [7]. Beyond its respiratory implications, this microorganism is capable of eliciting extra-respiratory complications, such as encephalitis, cold agglutinin hemolytic anemia mediated by IgM, and carditis. Additionally, Mycoplasma pneumoniae has been frequently associated with a variety of mucocutaneous manifestations, including maculopapular (Figure 3) or vesicular rashes, urticaria, erythema multiforme, Stevens-Johnson syndrome (SJS), and toxic epidermal necrolysis (TEN) [7].

In 2014, a distinct form of mucocutaneous presentation known as MIRM was introduced into the medical literature. MIRM can present differently than SJS, TEN, and other skin conditions, exhibiting unique characteristics such as significant mucosal involvement, which typically affects the urogenital and oral regions [8]. Some studies have indicated that 94% of patients with MIRM demonstrate oral manifestations, which can range from erosions to ulcerations, with severe cases presenting as hemorrhagic mucositis, as observed in our patient (Figure 2). Furthermore, ocular involvement has been reported as the second most common manifestation, often presenting as bilateral conjunctivitis (Figure 1). Genital involvement has been documented in 63% of patients with MIRM [9]. A new term has recently emerged in the literature: reactive infectious mucocutaneous eruptions (RIME). This term refers to all acute mucosa-dominant parainfectious eruptions and their associated causes. Numerous reports have identified a variety of microorganisms, including bacteria and viruses aside from Mycoplasma pneumoniae, that can lead to these acute mucocutaneous eruptions [10,11]. However, the use of this term is still under discussion. This situation highlights the need for a deeper understanding of the disease and its underlying mechanisms [12].

The diagnosis of MIRM is typically confirmed through a combination of clinical examination and serologic tests for Mycoplasma pneumoniae. Mucocutaneous symptoms in MIRM usually manifest after days to a few weeks from pulmonary symptoms, such as cough. It's worth noting that our patient did not display any respiratory symptoms or abnormalities during the chest examination and chest X-ray. This underscores the need for further research to improve the diagnosis of MIRM. Generally, MIRM has a favorable prognosis, with patients experiencing a good recovery rate without complications or recurrence, except in rare cases [13].

## Conclusions

This case report compellingly illustrates the urgent need to accurately differentiate MIRM from other serious skin conditions, such as SJS and TEN. Recognizing these distinctions is not just important; it fundamentally shapes the diagnosis and is crucial for determining the most effective treatment options available. In our case other specialties (rheumatologist, dermatologist, and ophthalmologist) involved in management of this patient and upon follow up patient's lesions healed. 

## References

[1] Krause DC, Balish MF (2001). Structure, function, and assembly of the terminal organelle of Mycoplasma pneumoniae. FEMS Microbiol Lett.

[2] Bébéar C, Pereyre S, Peuchant O (2011). Mycoplasma pneumoniae: susceptibility and resistance to antibiotics. Future Microbiol.

[3] Sánchez-Vargas FM, Gómez-Duarte OG (2008). Mycoplasma pneumoniae-an emerging extra-pulmonary pathogen. Clin Microbiol Infect.

[4] Narita M (2016). Classification of extrapulmonary manifestations due to Mycoplasma pneumoniae infection on the basis of possible pathogenesis. Front Microbiol.

[5] Canavan TN, Mathes EF, Frieden I, Shinkai K (2015). Mycoplasma pneumoniae-induced rash and mucositis as a syndrome distinct from Stevens-Johnson syndrome and erythema multiforme: a systematic review. J Am Acad Dermatol.

[6] (1990). Criteria for diagnosis of Behcet's disease. Lancet.

[7] Hammerschlag MR (2001). Mycoplasma pneumoniae infections. Curr Opin Infect Dis.

[8] Frantz GF, McAninch SA (2024). Mycoplasma mucositis. StatPearls.

[9] Lofgren D, Lenkeit C (2021). Mycoplasma pneumoniae-induced rash and mucositis: a systematic review of the literature. Spartan Med Res J.

[10] Vassallo C, Ruffo Di Calabria V, Isoletta E, Biscarini S, Di Filippo A, Brazzelli V (2021). Clinical and microbiological characteristics of reactive infectious mucocutaneous eruption: a case series of 5 patients. JAAD Case Rep.

[11] Ryder CY, Pedersen EA, Mancuso JB (2021). Reactive infectious mucocutaneous eruption secondary to SARS-CoV-2. JAAD Case Rep.

[12] Ramien ML (2021). Reactive infectious mucocutaneous eruption: Mycoplasma pneumoniae-induced rash and mucositis and other parainfectious eruptions. Clin Exp Dermatol.

[13] Poddighe D, Bruni P (2017). Mycoplasma pneumoniae-induced rash and mucositis (MIRM): an unusual mild skin rash associated with severe mucosal involvement. BMJ Case Rep.

